# Randomized phase II study of daily and alternate-day administration of S-1 for adjuvant chemotherapy in completely-resected stage I non-small cell lung cancer: results of the Setouchi Lung Cancer Group Study 1301

**DOI:** 10.1186/s12885-021-08232-6

**Published:** 2021-05-06

**Authors:** Norihito Okumura, Junichi Soh, Hiroyuki Suzuki, Masao Nakata, Toshiya Fujiwara, Hiroshige Nakamura, Makoto Sonobe, Takuji Fujinaga, Kazuhiko Kataoka, Kenichi Gemba, Masafumi Kataoka, Katsuyuki Hotta, Hiroshige Yoshioka, Keitaro Matsuo, Junichi Sakamoto, Hiroshi Date, Shinichi Toyooka

**Affiliations:** 1 Department of Thoracic Surgery, Kurashiki Central Hospital, 1-1-1 Miwa, Kurashiki, 710-8602 Japan; 2 Department of Surgery, Division of Thoracic Surgery, Kindai University Faculty of Medicine, 377-2 Ohno-Higashi, Osaka-, Sayama, Japan; 3 Department of Chest Surgery, Fukushima Medical University, 1 Hikarigaoka, Fukushima, Japan; 4 Department of General Thoracic Surgery, Kawasaki Medical School, 577 Matsushima, Kurashiki, Japan; 5 Depatment of Thoracic Surgery, Hiroshima City Hiroshima Citizens Hospital, 7-33 Moto-machi, Naka-ku, Hiroshima, Japan; 6 Division of General Thoracic Surgery, Tottori University Hospital, 36-1, Nishi-cho, Yonago, Japan; 7 Department of Thoracic Surgery, Kyoto University, 54 Shogoinkawara-cho, Sakyo-ku, Kyoto, Japan; 8 Department of General Thoracic Surgery, National Hospital Organization Nagara Medical Center, 1300-7 Nagara, Gifu, Japan; 9 Department of Thoracic Surgery, National Hospital Organization Iwakuni Clinical Center, 1-1-1 Atagomachi, Iwakuni, Japan; 10 Department of Respiratory Medicine, Chugoku Central Hospital, 148-13 Kamiiwanari, Miyuki-cho, Fukuyama, Japan; 11 Department of Surgery, Okayama Saiseikai General Hospital, 1-17-18 Ifuku-cho, Kita-ku, Okayama, Japan; 12 Center for Innovative Clinical Medicine, Okayama University Hospital, 2-5-1 Shikata-cho, Kita-ku, Okayama, Japan; 13 Department of Thoracic Oncology, Kansai Medical University Hospital, 2-3-1 Shinmachi, Hirakata, Osaka, Japan; 14 Division of Cancer Epidemiology and Prevention, Aichi Cancer Center Research Institute, 1-1 Kanokoden, Chikusa-ku, Nagoya, Japan; 15 Department of Preventive Medicine, Kyushu University Faculty of Medical Sciences, 3-1-1 Maidashi, Higashi-ku, Fukuoka, Japan; 16 Tokai Central Hospital, 4-6-2 Sohara Higashijima-cho, Kakamigahara, Japan; 17 Department of General Thoracic Surgery and Breast and Endocrinological Surgery, Okayama University Graduate School of Medicine, Dentistry, and Pharmaceutical Science, 2-5-1 Shikata-cho, Kita-ku, Okayama, Japan

**Keywords:** Non-small cell lung cancer, Adjuvant chemotherapy, S-1, Alternate-day administration

## Abstract

**Background:**

The aim of this multicenter, randomized phase II study was to analyze the feasibility and safety of alternate-day S-1, an oral fluoropyrimidine, for adjuvant chemotherapy in patients with completely resected pathological stage I (tumor diameter > 2 cm) non-small cell lung cancer (NSCLC).

**Methods:**

Patients were randomly assigned to receive adjuvant chemotherapy for 1 year comprising either alternate-day oral administration of S-1 (80 mg/m^2^/day) for 4 days a week (Group A) or a 2-week oral administration of S-1 (80 mg/m^2^/day) followed by 1 week of rest (Group B). The primary endpoint was feasibility, which was defined as the proportion of patients who completed the allocated intervention for 6 months with a relative dose intensity (RDI) of 70% or more.

**Results:**

Ninety-three patients were enrolled of whom 90 patients received S-1 treatment. Median follow-up was 66.9 months. The treatment completion rate based on an RDI of 70% or more for 6 months was 84.4% (95%CI; 70.5–93.5%) in group A and 64.4% (95%CI; 48.8–78.1%) in group B. There were no grade 4 adverse events in either group. Moderate or severe adverse events (grade 2 or grade 3) were significantly more frequent in group B (67%) compared with group A (29%, *P* = 0.001). The 5-year relapse-free survival rate was 87.0 and 80.9% for group A and B, respectively (*P* = 0.451). The 5-year overall survival rate for all patients (*n* = 93) was 100 and 89.4% for group A and B, respectively (*P* = 0.136).

**Conclusion:**

Alternate-day oral administration of S-1 for 1 year as adjuvant chemotherapy was demonstrated to be feasible with low toxicity in completely resected stage I (tumor diameter > 2 cm) NSCLC.

**Trial registration:**

Trial registration number: UMIN000011994.

Date of registration: 10/8/2013.

**Supplementary Information:**

The online version contains supplementary material available at 10.1186/s12885-021-08232-6.

## Background

Lung cancer is the most common cause of cancer-related death worldwide [1]. Surgery is considered to be the primary treatment modality for early stage non-small cell lung cancer (NSCLC) and the 5-year overall survival (OS) rates are 88.9 and 76.7% for pathological stage IA and IB NSCLC patients, respectively [2]. However, 15% of patients with stage IA NSCLC develop distant metastases even after complete resection [3].

A series of randomized controlled trials, including ANITA, JBR10, and CALGB9633, as well as the LACE meta-analysis, failed to demonstrate an OS benefit of platinum-based chemotherapy following complete surgical resection of pathological N0 NSCLC [4–7].

In Japan, however, a series of randomized phase III trials suggested a survival benefit for adjuvant chemotherapy with the oral drug uracil-tegafur (UFT) in patients with pathological N0 NSCLC following complete resection [8–10]. Meta-analysis by Hamada et al. revealed that UFT was beneficial for patients with a tumor size > 2 cm [11]. On the basis of these findings, the Japanese Lung Cancer Practice Guidelines, which were developed by the Japanese Society of Lung Cancer, recommended UFT therapy in completely resected N0 NSCLC patients with a tumor size > 2 cm for up to 2 years (https://www.haigan.gr.jp/modules/guideline/index.php?content_id=3). Furthermore, a recent randomized phase III trial conducted by our group demonstrated that no survival difference was observed between UFT versus paclitaxel plus carboplatin as adjuvant chemotherapy for completely resected stage IB to IIIB NSCLC [12].

S-1 (TS-1; Taiho Pharmaceutical Co., Ltd., Tokyo, Japan) is an oral fluoropyrimidine agent consisting of tegafur (a prodrug of 5-fluorouracil [5-FU]), gimeracil (an inhibitor of dihydropyrimidine dehydrogenase, which degrades fluorouracil), and oteracil, which inhibits the phosphorylation of fluorouracil in the gastrointestinal tract, thereby reducing the gastrointestinal toxic effects of fluorouracil, in a molar ratio of 1:0.4:1 [13]. S-1 was developed to improve the tumor-selective cytotoxicity of 5-FU, reduce gastrointestinal toxicity, and provide a higher antitumor effect compared with UFT. A phase II trial of S-1 monotherapy as first-line treatment for patients with advanced NSCLC resulted in a 22% response rate [14]. Regarding the adjuvant setting, S-1 adjuvant chemotherapy following curative surgery improved OS in gastric cancer or pancreatic cancer in phase III trials [15, 16]. Additionally, in a randomized phase III study, adjuvant chemotherapy with S-1 after curative treatment in patients with head and neck cancer revealed that OS was significantly greater in the S-1 group compared with that in the UFT group [17]. As for NSCLC, some phase II studies for resected NSCLC demonstrated the feasibility and efficacy of S-1 based adjuvant chemotherapy [18–21].

Recently, Kunitoh et al. reported the results of a phase III study that evaluated the efficacy of S-1 compared with UFT for post-operative adjuvant chemotherapy of node negative NSCLC. However, they were unable to demonstrate superiority of adjuvant S-1 therapy over UFT with respect to relapse-free survival (RFS) [22]. They suggested that one reason for the lack of superiority was that the outcomes of patients in the trial were exceedingly good for both arms and that future investigation should incorporate identification of, and be focued on, the high-risk populations for recurrence.

The original schedule for S-1 was a 4 week administration followed by a 2-week rest period for 1 year (conventional schedule) and the feasibility of administering S-1 according to this conventional schedule has been previously confirmed in patients with completely resected NSCLC [18, 23]. However, the discontinuation or dose reduction of S-1 often occurs because of adverse events during the conventional treatment schedule. To decrease the toxicity of S-1, a modified schedule has been used in which S-1 is administered for 2 weeks followed by a 1-week rest period (modified schedule) for patients receiving treatment according to the conventional schedule that experience severe toxicities [24].

A randomized scheduling feasibility study for S-1 has shown that the modified schedule appears to be more feasible compared with the conventional schedule in locoregionally advanced squamous cell carcinoma of the head and neck [25]. In our previous phase II study comparing the conventional and modified schedules of S-1 administration in patients with completely resected pathological stage IA (tumor diameter, 2–3 cm) NSCLC, we could not demonstrate a significant difference in feasibility between the two groups. However, we demonstrated that grade 3/4 toxicities were significantly less frequent in patients treated with the modified schedule compared with the conventional schedule despite equivalent survival for both groups [26]. As a result, the modified S-1 administration schedule has become more popular in clinical practice.

To further reduce the incidence of drug-induced adverse effects and improve the feasibility of administration, an alternate-day S-1 schedule has recently been investigated. The alternate-day regimen of S-1 was safer and more tolerable compared with the daily regimen in patients with gastric, pancreatic, and head and neck cancer [27–29]. In recent reports, an alternate-day administration of S-1 was also demonstrated to be a potentially safe treatment regimen for elderly patients with advanced NSCLC [30]. To our knowledge, however, there are no reports of prospective studies using an alternate-day S-1 administration of adjuvant chemotherapy for completely resected NSCLC.

Therefore, we conducted a multicenter, randomized phase II trial comparing the feasibility and safety of alternate-day administration of S-1 versus daily administration for 2 weeks followed by a 1-week rest period as adjuvant chemotherapy in completely resected stage I (tumor diameter > 2 cm) NSCLC.

## Methods

### Patients

Patients who met all the following eligibility criteria and none of the exclusion criteria which are listed in Online Resource 1 were enrolled in this study. The eligibility criteria were as follows: (i) completely resected NSCLC, pathological stage I (according to the Union Internationale Countre le Cancer [UICC] seventh TNM edition) [31] with a tumor diameter > 2 cm, (ii) within 4–6 weeks following surgical resection (lobectomy and larger lung resection) with complete lymph node dissection (ND2a and more extensive in principle), (iii) patients who were able to begin the protocol treatment within 8 weeks after surgical resection, (iv) no prior chemotherapy or radiotherapy, (v) age 20–74 years, (vi) an Eastern Cooperative Oncology Group (ECOG) performance status (PS) of 0 or 1, (vii) adequate organ function [leukocytes ≥3000/μl, neutrophils ≥1500/μl, platelets ≥100,000/μl, hemoglobin ≥9.0 g/dl, total bilirubin ≤1.5 mg/dl, aspartate aminotransferase (AST) and alanine aminotransferase (ALT) each ≤100 IU/l, creatinine clearance ≥40 ml/min, PaO_2_ ≥ 60 mmHg] and (viii) written informed consent.

All patients provided written informed consent before enrollment in the study.

### Treatment and follow-up

**Fig. 1:**
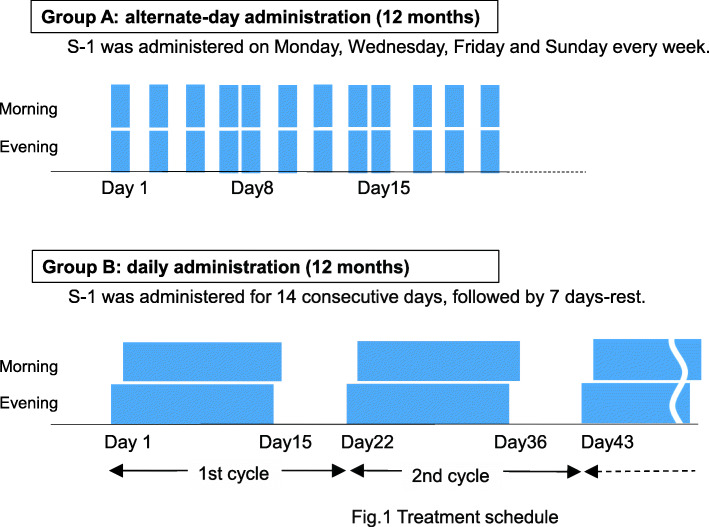
Treatment schedule

The randomization was performed centrally at the Department of Preventive Medicine, Kyushu University Faculty of Medical Sciences, Fukuoka, Japan, with the following stratification factors: institution, histology (squamous cell carcinoma or non-squamous cell carcinoma), pathological stage [stage IA (tumor diameter > 2 cm) or stage IB], and epidermal growth factor (EGFR) mutational status (positive or negative). The patients received S-1 orally twice daily. The dose was 80 mg/body/day when the body surface area was < 1.25 m^2^, 100 mg/body/day for 1.25–1.50 m^2^, and 120 mg/body/day for > 1.50 m^2^. S-1 was randomly administered on alternate days for 4 days (Monday, Wednesday, Friday and Sunday) 1 week (group A) or daily 2 weeks followed by a 1-week rest period (group B). These cycles were repeated every week (group A) or every 3 weeks (group B) for 1 year after the start of oral administration (Fig. 1). The details of the criteria for discontinuation and restart of S-1 administration, the manner of dose reduction, and the criteria for cessation of the treatment protocol are provided in Online Resource 2, 3, 4 and 5.

As for baseline evaluations, medical history, smoking history, physical examination, operation date, p-TNM status, tumor histology (squamous cell carcinoma or non-squamous cell carcinoma), comorbidity, and laboratory analyses were included. The details of the follow-up assessments are provided in Online Resource 6. Toxicity was graded according to the Common Terminology Criteria for Adverse Events (CTCAE), version 4.0.

### Study design and statistical analysis

The study was designed as a multicenter randomized phase II study, conducted in accordance with the Declaration of Helsinki, and registered with the UMIN Clinical Trial Registry, UMIN000011994. This study adheres to CONSORT guidelines. The study protocol was approved by the institutional review board of each participating institution. All of the study data were managed by the Setouchi Lung Cancer Group (SLCG) 1301 data center at a nonprofit organization, the Epidemiological and Clinical Research Information Network (ECRIN), Kyoto, Japan. The primary endpoint of this study was feasibility, which was defined as the proportion of patients who completed the allocated intervention for 6 months with 70% or more of relative dose intensity (RDI). RDI was defined as the rate between the actual total administration dose and the planned total administration dose. Treatment completion rates under the conditions described above at 9 months and at 12 months after the initiation of the treatment protocol were also calculated.

This study was designed according to a randomized phase II selection design [32]. We assumed that the threshold 6-month treatment completion rate for the current protocol in both groups was 60%. The decision criteria for the primary endpoint were as follows: 1) If the 6-month treatment completion rate is 60% or less in both groups, the protocol treatments for both groups is not considered promising. 2) If the 6-month treatment completion rate of one group is more than 60% and exceeds that of another group by at least 15%, the regimen for the group in which the completion rate was higher is considered promising and selected for further phase III study. 3) If the 6-month treatment completion rate of one group is more than 60% and exceeds that of another group by 15% or less, the comparison between the two groups should not be performed with the treatment completion rate. According to the design for assuring 90% probability for selecting the best study arm, provided that the true expected completion rate exceeded that of another arm by at least 15%, we estimated that the required number of patients would be 37 for each arm. Finally, the sample size was set to 90 considering the potential for patient drop-out because of ineligibility.

The secondary endpoints were toxicity, RFS, and OS. Patients who discontinued the treatment protocol because of tumor recurrence or other complications unrelated to S-1 were treated as censored cases. A final analysis of survival time is expected to be done 5 years after the last enrollment.

Significance differences between the categorized groups were compared using the Fisher’s exact test or the Mann-Whitney test. Univariate analysis of OS and RFS was performed using the Kaplan-Meier method with log-rank testing. We defined *P* < 0.05 as the threshold for statistical significance. All the statistical analyses were executed using SPSS Statistics ver25 software (IBM, NY, USA).

## Results

### Patient characteristics

**Fig. 2:**
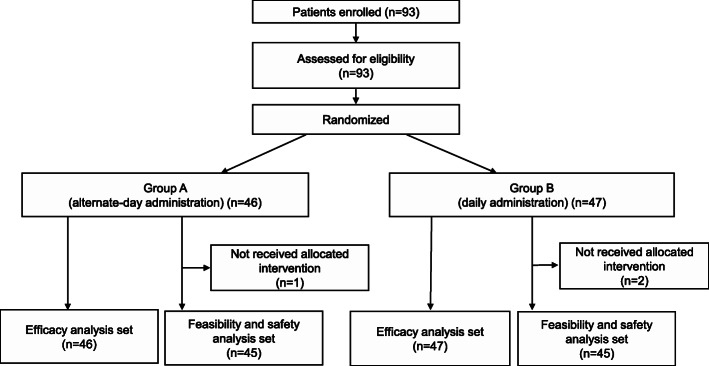
CONSORT diagram for the current study

Ninety-three patients were enrolled in this trial from 20 institutions in Japan from November 2013 to May 2015. Three patients refused the protocol treatment and 90 patients received allocated intervention (45 in group A and 45 in group B) (Fig. 2). The baseline characteristics of the enrolled patients are summarized in Table 1. Fifty-two patients (55.9%) were men and the median age was 67 years old. Eighty-eight (94.6%) patients had non-squamous cell carcinoma histology and 42 (45.2%) patients were pathological Stage IB.

**Table 1:** Patient characteristics

Variables		Total(n = 93)	Group A(*n* = 46)	Gropu B(B = 47)	*P* value
Age, y	Median (range)	67 (40–74)	67 (49–74)	66 (40–74)	0.489
Sex	Male	52	28	24	0.406
	Female	41	18	23	
Smoking	Present or former smoker	52	28	24	0.406
	Non-smoker	41	18	23	
ECOG PS	0	79	38	41	0.574
	1	14	8	6	
Histology	Sq	5	2	3	1.000
	Non-Sq	88	44	44	
Pathological stage	IA (T > 2 cm)	51	24	27	0.679
	IB	42	22	20	
EGFR-mutation	Positive	44	21	23	0.836
	Negative	49	25	24	

*ECOG PS* Eastern Cooperative Oncology Group perfprmance status, *Sq* Squamous cell carcinoma, *Non-Sq* Non-squamous cell carcinoma, *EGFR* Epidermal growth factor receptor

### Feasibility

During the 1-year treatment course, 35.6% of the patients in group A and 15.6% of the patients in group B received S-1 administration according to the planned schedule and completed the initial dose without requiring a dose reduction (*P* = 0.052). Treatment discontinuation rates for 1 year were 26.7 and 44.4% in group A and B, respectively (*P* = 0.123). Treatment was discontinued because of adverse events or toxicity in 5 (11.1%) and 12 (26.7%) patients in group A and B, respectively (*P* = 0.104). The means of the RDI at 1 year were 78.7% (SD, 25.6%) in group A and 59.4% (SD, 32.1%) in group B, respectively (*P* = 0.001) (Table 2).

**Table 2:** Treatment discontinuation and relative dose intensity

	Group A (*n* = 45)	Group B (*n* = 45)	*P* value
Treatment discontinuation	12 (26.7%)	20 (44.4%)	0.123
*Reason for discontinuation*			
Toxicity or adverse event	5 (11.1%)	12 (26.7%)	0.104
Recurrence	2 (4.4%)	1 (2.2%)	1.000
2nd malignancy	1 (2.2%)	1 (2.2%)	1.000
Patient refusal	3 (6.7%)	3 (6.7%)	1.000
Investigator discision	1 (2.2%)	2 (2.2%)	1.000
Others	0 (0%)	1 (2.2%)	1.000
Relative dose intensity at 1 year			
Mean	78.7%	59.4%	0.001
Standard deviation	25.6%	32.1%	

Treatment completion rates at 6 months with RDI values more than 70% were 84.4% [95% confidence interval (CI), 70.5–93.5%] for group A and 64.4% (95%CI, 48.8–78.1%) for group B (*P* = 0.052). Treatment completion rates at 9 and 12 months in both groups are also shown in Table 3. The treatment completion rate at 6 months with an RDI value of 70% or more in group A was greater than 60% and it exceeded that of group B by more than 15%. According to the established decision criteria, these results indicated that the regimen for group A was promising and it should be selected for a phase III study of adjuvant chemotherapy in completely resected stage I NSCLC.

**Table 3:** Treatment completion rate (Relative dose intensity ≥ 70%)

	Group A (*n* = 45)	Group B (*n* = 45)	*P* value
6 months	38 (84.4%) (95%CI; 70.5–93.5%)	29 (64.4%) (95%CI; 48.8–78.1%)	0.052
9 months	34 (75.6%) (95%CI; 60.5–87.1%)	26 (57.8%) (95%CI; 42.2–72.3%)	0.117
12 months	31 (68.9%) (95%CI; 53.4–81.8%)	20 (44.4%) (95%CI; 29.6–60.0%)	0.033

*CI* Confidence interval

### Toxicity

A summary of the adverse events is shown in Table 4. Toxicities were generally well-tolerated in both groups and there were no grade 4 adverse events for any patient. There were also no grade 3 or worse hematological adverse events in either group. The incidence of an adverse event of any grade was 100 and 89% in group B and A, respectively (*P* = 0.056). Moderate or severe adverse events (grade 2 or grade 3) were significantly more frequent in group B (67%) compared with group A (29%, *P* = 0.001). There were no treatment-related deaths in either group during treatment.

**Table 4:** adverse event

Adverse event	Group A (*n* = 45)					Group B (*n* = 45)					*P* value	
	G1	G2	G3	Any grade (%)	G2/G3 (%)	G1	G2	G3	Any grade (%)	G2/G3 (%)	Any grade	G2/G3
Any adverse event	27	8	5	40 (89%)	13 (29%)	15	24	6	45 (100%)	30 (67%)	0.056	0.001
Anemia	13	1	0	14 (31%)	1 (2%)	21	2	0	23 (51%)	2 (4%)	0.086	1.000
Leukopenia	7	2	0	9 (20%)	2 (4%)	12	2	0	14 (31%)	2 (4%)	0.334	1.000
Neutropenia	9	1	0	10 (22%)	1 (2%)	7	0	0	7 (16%)	0 (0%)	0.591	1.000
Thrombocytopenia	7	0	0	7 (16%)	0 (0%)	15	0	0	15 (33%)	0 (0%)	0.085	****
Elevation of AST	12	1	1	14 (31%)	2 (4%)	12	0	0	12 (27%)	0 (0%)	0.816	0.494
Elevation of ALT	12	2	0	14 (31%)	2 (4%)	13	1	0	14 (31%)	1 (2%)	1.000	1.000
Elevation of LDH	5	0	0	5 (11%)	0 (0%)	10	0	0	10 (22%)	0 (0%)	0.258	****
Elevation of bilirubin	15	1	0	16 (36%)	1 (2%)	14	6	1	21 (47%)	7 (16%)	0.392	0.058
Elevation of creatinine	3	0	0	3 (7%)	0 (0%)	5	0	0	5 (11%)	0 (0%)	0.714	****
Anorexia	8	0	1	9 (20%)	1 (2%)	17	4	1	22 (49%)	5 (11%)	0.007	0.203
Nausea	12	0	0	12 (27%)	0 (0%)	13	4	0	17 (38%)	4 (9%)	0.367	0.117
Vomiting	4	0	0	4 (9%)	0 (0%)	6	1	0	7 (16%)	1 (2%)	0.522	1.000
Diarrhea	5	3	0	8 (18%)	3 (7%)	10	2	1	13 (29%)	3 (7%)	0.319	1.000
Stomatitis	9	0	0	9 (20%)	0 (0%)	9	2	1	12 (27%)	3 (7%)	0.619	0.242
Fatigue	4	0	0	4 (9%)	0 (0%)	5	1	0	6 (13%)	1 (2%)	0.739	1.000
Skin symptoms	4	0	1	5 (11%)	1 (2%)	13	6	1	20 (44%)	7 (16%)	0.001	0.058
Keratinitis/conjunctivitis	0	0	0	0 (0%)	0 (0%)	5	2	0	7 (16%)	2 (4%)	0.012	0.494

**** incalculable

The main adverse events were hematological, gastrointestinal, and cutaneous symptoms. Among these, anorexia, skin symptoms, and keratitis/conjunctivitis were more frequent in group B (49, 44 and 16%,repectively) compared with group A (20%; *P* = 0.007, 11%; *P* = 0.001 and 0%,; *P* = 0.012, respectively).

### Survival

**Fig. 3:**
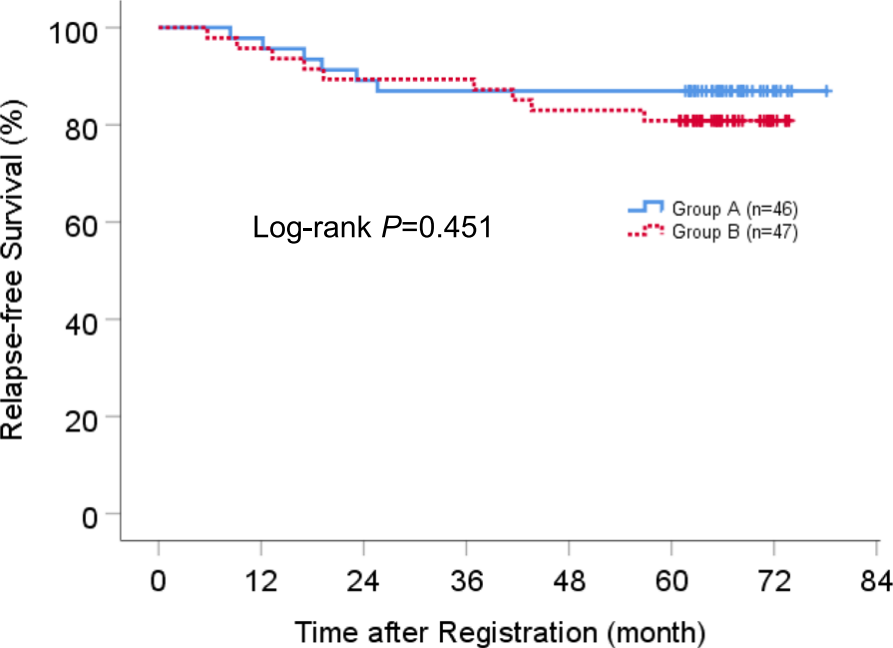
Kaplan-Meier estimates of relapse-free survival for all patients

**Fig. 4:**
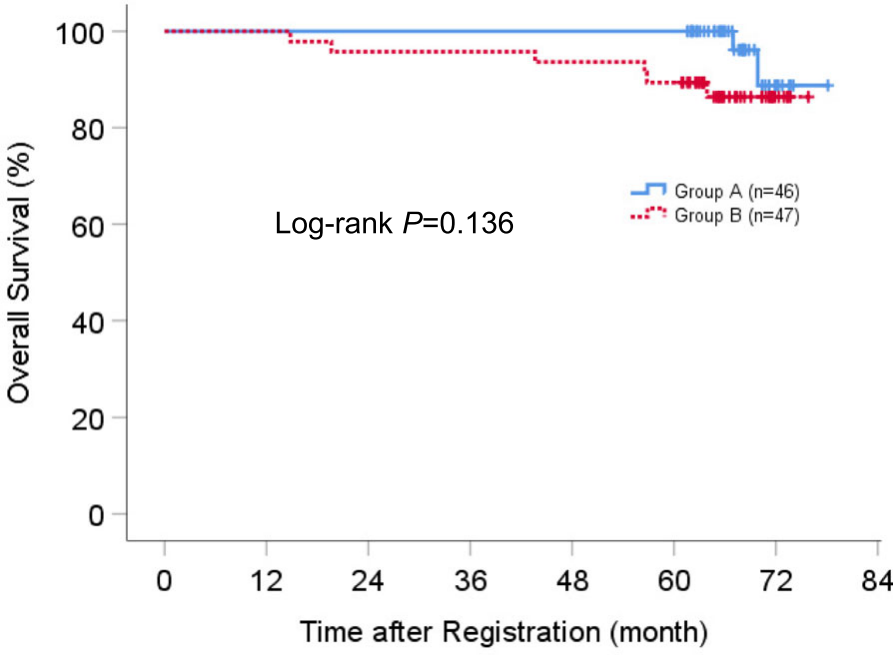
Kaplan-Meier estimates of overall survival for all patients

The median follow-up time was 66.9 months (range, 14.8 to 78.0 months), at which point patients who received the protocol treatment (*n* = 90) and all patients (*n* = 93) had been monitored for at least 5 years. Survival analyses were performed based on an intention to treat. The 5-year RFS rate for all patients (*n* = 93) was 87.0 and 80.9% for group A and B, respectively (*P* = 0.451) (Fig. 3). The 5-year OS rate for all patients (*n* = 93) was 100 and 89.4% for group A and B, respectively (*P* = 0.136) (Fig. 4). Regarding the impact of EGFR gene status on survival, there were no significant differences in OS and RFS between EGFR mutant and wild-type cases among the total population (see Online Resource 7, 8).

## Discussion

The cell cycle period of normal cells is shorter than that of tumor cells (0.5–1 day versus 3–7 days, respectively) and the duration of the S-phase in tumor cells is ≥1 day. 5-FU exhibits activity toward S-phase cells by inhibiting DNA synthesis and cell proliferation. Shirasaka et al. recommended the use of an alternate-day schedule for S-1 administration instead of daily use based on the observation that the former would provide time for the recovery of normal epithelial cells in the gastrointestinal tract and bone marrow while still maintaining an anticancer effect [33]. Using gastric cancer cell lines in vitro and in vivo, a previous study showed that alternate-day S-1 administration was associated with lower toxicity while yielding a similar antitumor effect compared with standard daily administration [34]. Moreover, a retrospective study showed that alternate-day S-1 administration was associated with reduced adverse events compared with standard daily S-1 administration in patients with advanced gastric cancer [35]. In a randomized phase II trial for patients with advanced NSCLC, Suzuki et al. demonstrated that the alternate-day administration of S-1 was less toxic and did not compromise its therapeutic effectiveness compared with the standard daily administration of S-1 [36].

In the present study, we compared alternate-day administration of S-1 with daily administration for 2 weeks followed by a 1-week rest in an adjuvant setting for completely resected stage I (T > 2 cm) NSCLC. In an analysis of a phase III study of postoperative gastric cancer [15], when the protocol completion cases were divided into two groups according to compliance with S-1 administration, the 5-year survival curves overlapped for patients with ≥90% RDI and patients with 70 to < 90% RDI (in-house experimental data; Taiho Pharmaceutical). Therefore, we concluded that an RDI of 70% provides sufficient adjuvant chemotherapy for NSCLC with S-1, similar to gastric cancer. A treatment completion rate of 6 months with an RDI of 70% or more in the alternate-day administration group was over 60% (86.7%) and exceeded that in the daily administration group by more than 15% (22.3%). On the basis of this result, an alternate-day administration of S-1 appears to be feasible and promising for adjuvant chemotherapy in completely resected stage I NSCLC.

Toxicities were generally well-tolerated in the current study and there was no grade 4 or worse adverse event in either group. However, moderate or severe adverse events (grade 2 or grade 3) were significantly more frequent in the daily administration group (67%) compared with the alternate-day administration group (29%, *P* = 0.001). Moreover, anorexia, skin, and eye symptoms were significantly more frequent in the daily administration group compared with the alternate-day group, indicating that the alternate-day administration may be more tolerable.

In an adjuvant setting, low toxicity is a particularly important factor because postoperative frail patients are likely to be overcome with toxicity and treatment discontinuation resulting from toxicity may attenuate the effectiveness of adjuvant chemotherapy. As a result, alternate-day administration of S-1 would be a good candidate for adjuvant chemotherapy. As for alternate-day administration of S-1, fewer studies on adjuvant chemotherapy have been reported compared with that of chemotherapy for advanced cases. Prospective studies of alternate-day S-1 as adjuvant chemotherapy for gastric cancer and head and neck cancer demonstrated improved feasibility, low toxicity, and efficacy [28, 37]. The current study demonstrated that alternate-day administration of S-1 for adjuvant chemotherapy in NSCLC was also feasible and exhibited low toxicity with an acceptable survival rate.

In the phase III study by Kunitoh et al., S-1 was not superior to the efficacy of UFT in the same population as our study. They administered S-1 daily for 2 weeks followed by a 1-week rest for 1 year. The dose reduction rate resulting from treatment toxicity was 40.3% in the S-1 arm compared with 20.1% in the UFT arm and there were three treatment-related deaths in the S-1 arm [22]. If an alternate-day S-1 administration is adopted, the feasibility and safety of the S-1 arm may be improved while enhancing the effectiveness of the adjuvant chemotherapy. Therefore, our results that demonstrated to be feasible with low-toxicity of alternate-day S-1 administration as adjuvant chemotherapy in resected stage I NSCLC is considered to have a significant clinical implication irrespective of the results of the phase III study by Kunitoh et al.

In addition, the primary endpoint of the Kunitoh study, 5-year RFS, was 79.5% in the S-1 arm compared with 79.4% in the UFT arm. The treatment duration was 1 and 2 years in the S-1 and UFT arms, respectively. Considering a similar efficacy for both arms, short duration adjuvant chemotherapy with S-1 may be advantageous compared with UFT therapy.

This study had some limitations. The weaknesses of our study design included the lack of pill counts. Drug compliance may be an important challenge in this study. In addition, information of driver mutation other than EGFR or programmed cell death-ligand 1 (PD-L1) were not collected in this trial. We also did not investigate outcome according to stratification by patient age. A previous study of adjuvant chemotherapy with S-1 according to a conventional daily schedule demonstrated that the RDI of patients over 65 years old was significantly lower compared with that of the other patients. Additionally, the conventional daily administration of S-1 was not likely to be feasible in elderly patients with completely resected NSCLC [22]. However, our group is now conducting a randomized phase II trial to confirm the advantage of alternate-day S-1 administration compared with daily S-1 administration as adjuvant chemotherapy for elderly NSCLC patients (UMIN000007819).

## Conclusion

Alternate-day oral administration of S-1 was demonstrated to be feasible with low-toxicity as adjuvant chemotherapy in completely resected stage I (tumor diameter > 2 cm) NSCLC. These results are promising and warrant a subsequent phase III study of alternate-day administration of S-1 compared with the standard care for these patients, which should be incorporate identification of, and be focued on, the high-risk populations for recurrence according to the results of JCOG0707.

## Supplementary Information

**Additional file 1.** Online Resource 1. The exclusion criteria.

**Additional file 2.** Online Resource 2. The criteria for discontinuation and restant of S-1 administration.

**Additional file 3.** Online Resource 3. Criteria for dose reduction.

**Additional file 4.** Online Resource 4. Method for dose reduction of S-1.

**Additional file 5.** Online Resource 5. The criteria for cessation of the protocol treatment.

**Additional file 6.** Online resourse 6. Follow-up assessment.

**Additional file 7.** Online Resource 7. Kaplan-Meier estimates of overall survival.

**Additional file 8.** Online Resource 8. Kaplan-Meier estimates of relapse-free survival.

## Data Availability

Data and materials of this work are available from the corresponding author on reasonable request.

## Abbreviations

NSCLC: Non-small cell lung cancer
RDI: Relative dose intensity
CI: Confidence interval
SD: Standard deviation
UICC: Union Internationale Countre le Cancer
ECOG: Eastern Cooperative Oncology Group
PS: Performance status
AST: Aspartate aminotransferase
ALT: Alanine aminotransferase
EGFR: Epidermal growth factor
CTCAE: The Common Terminology Criteria for Adverse Events
RFS: Relapse-free survival
OS: Overall survival
DNA: Deoxyribonucleic acid
