# Supplementary material for: Randomized phase II study of daily and alternate-day administration of S-1 for adjuvant chemotherapy in completely-resected stage I non-small cell lung cancer: results of the Setouchi Lung Cancer Group Study 1301
Source: BMC Cancer. 2021 May 6;21:506. doi: 10.1186/s12885-021-08232-6 (PMC8101150; doi:10.1186/s12885-021-08232-6)
Supplement: Supplementary file 1 — Additional file 1. Online Resource 1. The exclusion criteria. [file 12885_2021_8232_MOESM1_ESM.docx]

Online Resource 1. The exclusion criteria

1. Contraindication of S-1
2. Serious infectious disease, uncontrolled diabetes mellitus, ileus, watery diarrhea, or other diseases interfering with S-1 treatment
3. History of serious hypersensitivity to drugs
4. Acute myocardial infarction within 6 months
5. Interstitial pneumonia or obvious interstitial shadow on chest X-ray
6. Active concomitant malignancy
7. Pregnancy or lactation
8. Psychiatric disease
9. Administration of other pyrimidine fluoride drugs
10. Administration of warfarin and dabigatran etexilate
11. Other inadequate conditions as determined by the attending physician.
