# Supplementary material for: Randomized phase II study of daily and alternate-day administration of S-1 for adjuvant chemotherapy in completely-resected stage I non-small cell lung cancer: results of the Setouchi Lung Cancer Group Study 1301
Source: BMC Cancer. 2021 May 6;21:506. doi: 10.1186/s12885-021-08232-6 (PMC8101150; doi:10.1186/s12885-021-08232-6)
Supplement: Supplementary file 5 — Additional file 5. Online Resource 5. The criteria for cessation of the protocol treatment. [file 12885_2021_8232_MOESM5_ESM.docx]

Online Resource 5. The criteria for cessation of the protocol treatment

1. Severe toxicities or complication which preclude the continuity of protocol treatment
2. Protocol treatment could not restart due to toxicities until more than 28 days after the last adminstration of S-1
3. Relapse or 2nd malignancy
4. Patient’s refusal
5. Other inadequate conditions as judged by the attending physician
