# Supplementary figures and images for: Randomized phase II study of daily and alternate-day administration of S-1 for adjuvant chemotherapy in completely-resected stage I non-small cell lung cancer: results of the Setouchi Lung Cancer Group Study 1301
Source: BMC Cancer. 2021 May 6;21:506. doi: 10.1186/s12885-021-08232-6 (PMC8101150; doi:10.1186/s12885-021-08232-6)

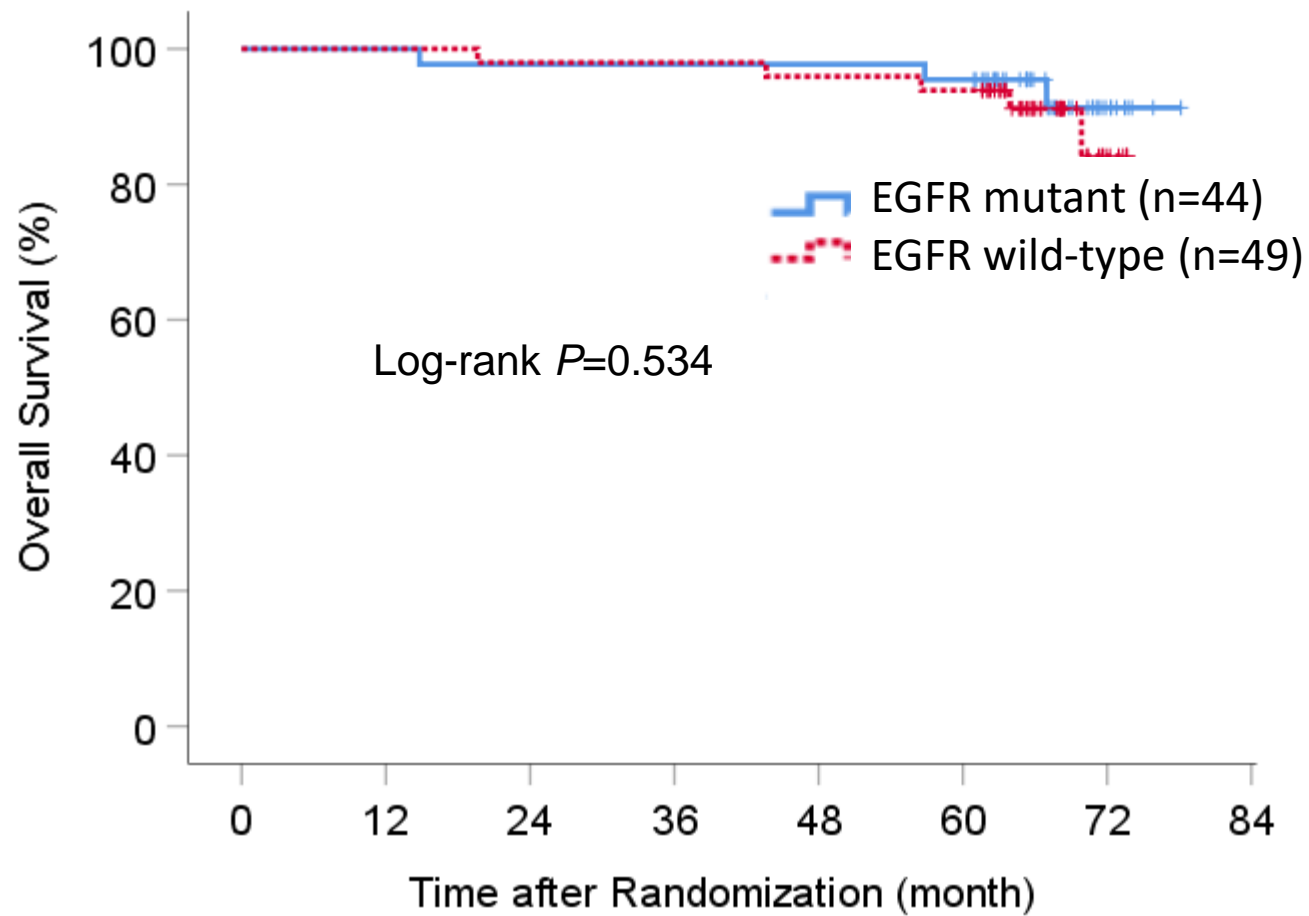

Online Resource 7. Kaplan-Meier estimates of overall survival

Supplement: Supplementary file 7 — Additional file 7. Online Resource 7. Kaplan-Meier estimates of overall survival. [file 12885_2021_8232_MOESM7_ESM.pdf]

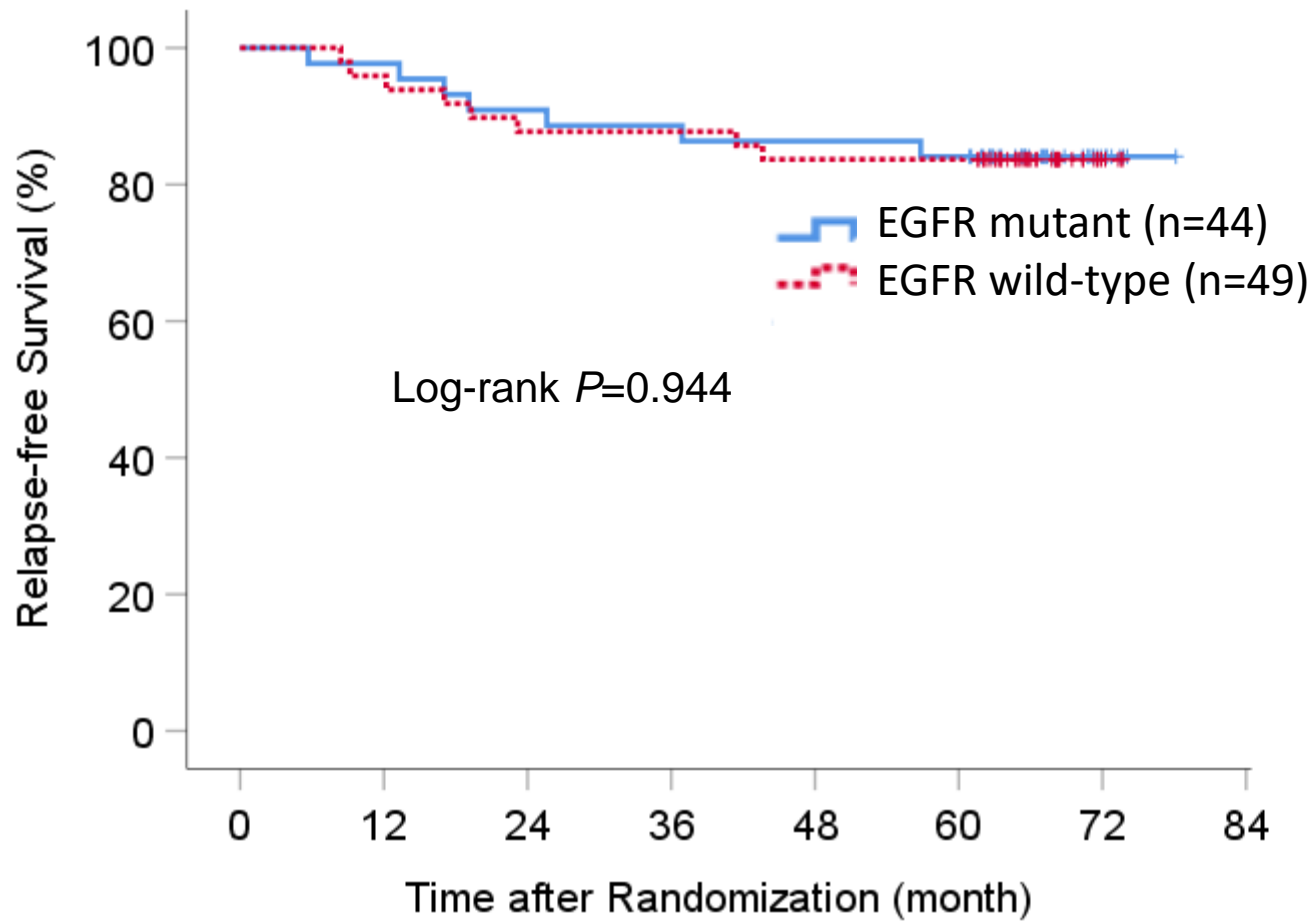

Online Resource 8. Kaplan-Meier estimates of relapse-free survival

Supplement: Supplementary file 8 — Additional file 8. Online Resource 8. Kaplan-Meier estimates of relapse-free survival. [file 12885_2021_8232_MOESM8_ESM.pdf]
